# A review of antidiabetic active thiosugar sulfoniums, salacinol and neokotalanol, from plants of the genus *Salacia*

**DOI:** 10.1007/s11418-021-01522-0

**Published:** 2021-04-26

**Authors:** Toshio Morikawa, Kiyofumi Ninomiya, Genzoh Tanabe, Hisashi Matsuda, Masayuki Yoshikawa, Osamu Muraoka

**Affiliations:** 1 Pharmaceutical Research and Technology Institute, Kindai University, 3-4-1 Kowakae, Higashi-osaka, Osaka, 577-8502 Japan; 2 Antiaging Center, Kindai University, 3-4-1 Kowakae, Higashi-osaka, Osaka, 577-8502 Japan; 3 Faculty of Pharmacy, Kindai University, 3-4-1 Kowakae, Higashi-osaka, Osaka, 577-8502 Japan; 4 Kyoto Pharmaceutical University, 1 Shichono-cho, Misasagi, Yamashina-ku, Kyoto, 607-8412 Japan; 5 Present Address: School of Pharmacy, Shujitsu University, 1-6-1 Nishigawara, Naka-ku, Okayama, Okayama 703-8516 Japan

**Keywords:** *Salacia*, Salacinol, Neokotalanol, *α*-glucosidase inhibitor, Diabetes, Functional food

## Abstract

**Abstract:**

During our studies characterizing functional substances from food resources for the prevention and treatment of lifestyle-related diseases, we isolated the active constituents, salacinol (**1**) and neokotalanol (**4**), and related thiosugar sulfoniums, from the roots and stems of the genus *Salacia* plants [Celastraceae (Hippocrateaceae)] such as *Salacia reticulata* Wight, *S. oblonga* Wall., and *S. chinensis* L., and observed their antidiabetic effects. These plant materials have been used traditionally in Ayurvedic medicine as a specific remedy at the early stage of diabetes, and have been extensively consumed in Japan, the United States, and other countries as a food supplement for the prevention of obesity and diabetes. Here, we review our studies on the antidiabetic effects of plants from the genus *Salacia*, from basic chemical and pharmacological research to their application and development as new functional food ingredients.

**Graphic abstract:**

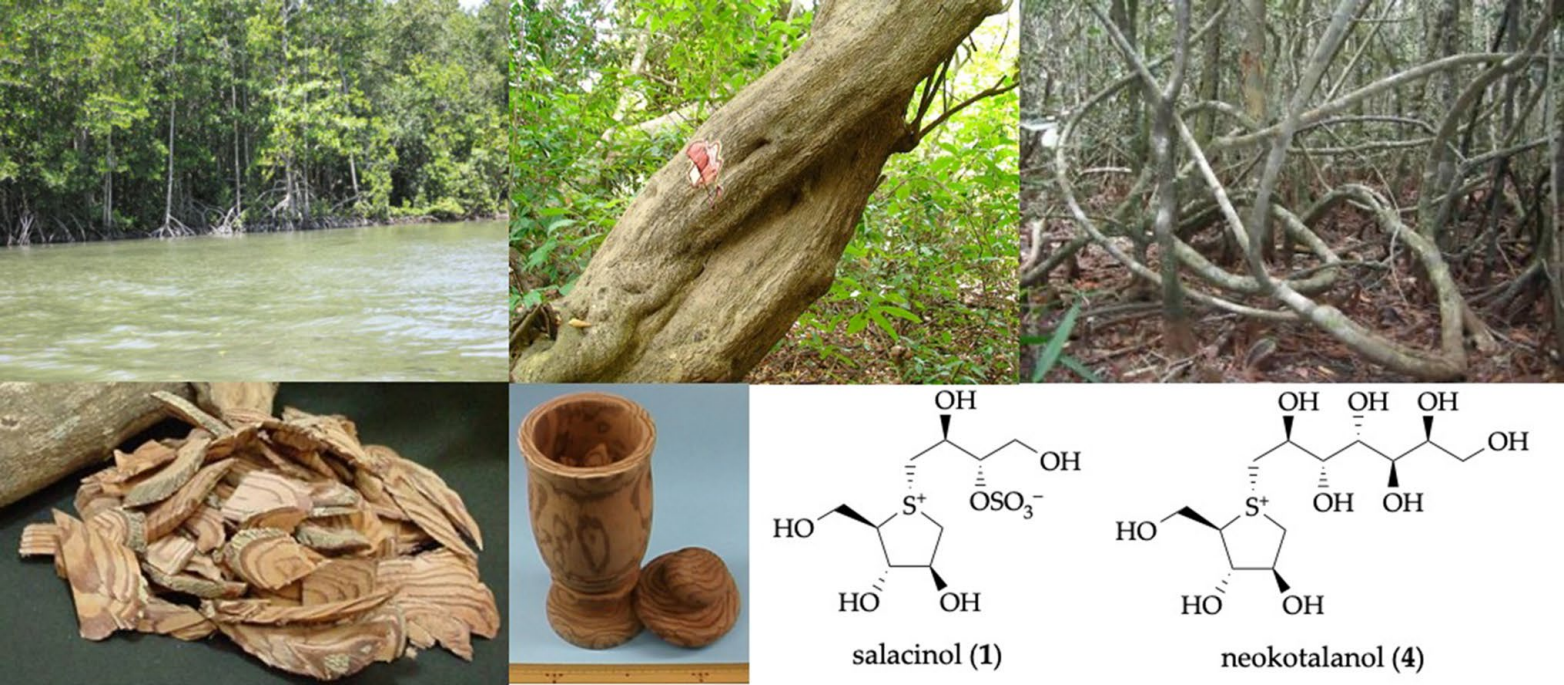

## Introduction

**Fig. 1:**
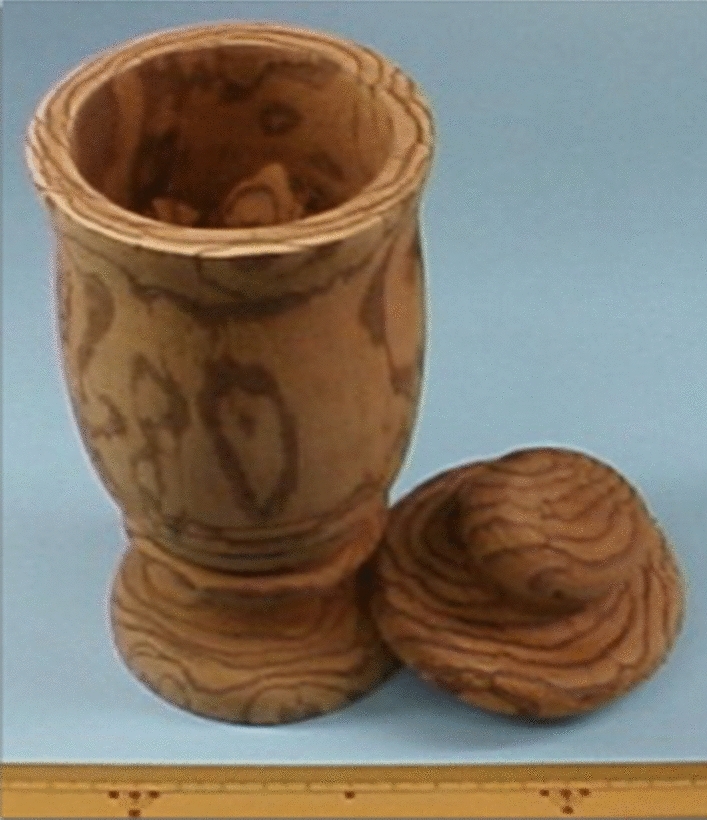
A mug made from the roots and stems of *Salacia reticulata* in Sri Lanka

**Fig. 2:**
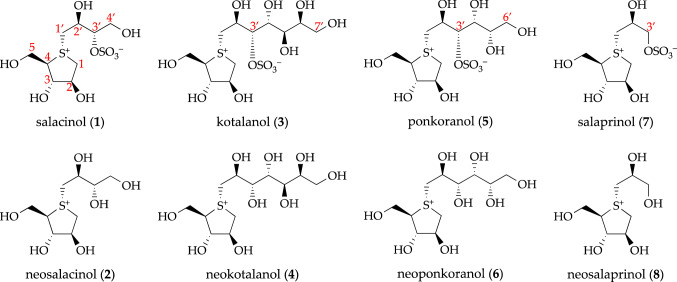
Structures of salacinol (**1**), neosalacinol (**2**), kotalanol (**3**), neokotalanol (**4**), and related constituents (**5**–**8**)

Plants of the genus *Salacia,* classified as the Celastraceae (Hippocrateaceae) family [1], are widely distributed in Sri Lanka, India, Southeast Asia (*e.g.,* Thailand and Indonesia), and in torrid zone areas, such as Brazil [2–6]. According to The Plant List (www.theplantlist.org, accessed on March 16, 2021), 481 plants from *Salacia* genus, including *S. reticulata* Wight (an unresolved name), *S. oblonga* Wall. (an unresolved name, synonym of *Comocladia serrata* Blanco), and *S. chinensis* L. (an accepted name, synonyms of *S. prinoides* Willd. DC. and *Tontelea prinoides* Willd.), are recorded [1]. These *Salacia* plants are termed locally as “Kotala himbutu” in Singhalese for *S. reticulata*; “Chundan” in Tamil and “Ponkoranti” in Malayalam for *S. oblonga*; and “Kam Phaeng Chetchan” in Thai for *S. chinensis* [2, 7]. Their roots and stems have been used extensively for thousands of years in traditional medicines for the treatment of rheumatism, gonorrhea, and skin diseases. In the Ayurvedic system [8–10] and in Thai traditional medicine [11], they have also been used as a remedy at the early stage of diabetes. Traditionally, in Sri Lanka, aqueous extract was prepared by storing water overnight in mugs made from the root and stem parts of *S. reticulata* (Fig. 1). Throughout the course of our studies characterizing functional substances from food resources for the prevention and treatment of lifestyle-related diseases, our research group has focused on the antidiabetic effects of plants from the genus *Salacia* since the mid-1990s. Before we began our research, data on the in vivo hypoglycemic activities of extracts from *S. reticulata* [12, 13], *S. oblonga* [14], and *S. chinensis* [15] had been reported. However, at that time, the active constituents, and the mechanisms underlying the antidiabetic effects of plants from the genus *Salacia* had not yet been characterized. Here, we review our studies on the antidiabetic effects of plants from the genus *Salacia*, from basic chemical and pharmacological research to their application and development as new functional food ingredients. Particularly, we focus on and describe active constituents with antidiabetic activity, including salacinol (**1**), neosalacinol (**2**), kotalanol (**3**), neokotalanol (**4**), and related analogs (**5**–**8**), which are unique thiosugar sulfonium constituents with a novel class of *α*-glucosidase inhibitors from plants of the *Salacia* genus (Fig. 2).

## Search for active antidiabetic constituents

### Suppressive effects of the methanol extract from the roots and stems of *S. reticulata* on postprandial blood glucose elevation in sugar-loaded rats

According to “9th Edition of The International Diabetes Federation (IDF) Atlas”, diabetes is one of the fastest growing global health emergencies of the twenty-first century. In 2019, an estimated 463 million individuals had diabetes, and this number is projected to reach 578 million by 2030, and 700 million by 2045 [16]. Type 2 diabetes mellitus, or non-insulin-dependent diabetes mellitus, is a chronic metabolic disorder characterized by symptoms such as hyperglycemia, insulin resistance, and relative insulin deficiency [17]. Chronic hyperglycemia can lead to long-term complications, such as cardiovascular and renal disorders, retinopathy, and poor blood flow. The development of type 2 diabetes mellitus can be prevented or delayed in individuals with impaired glucose tolerance by implementing lifestyle changes or through the use of therapeutic agents [18]. Through wide in vivo screening trials, we have identified extracts and their constituents isolated from several natural resources, including *Kochia scoparia* [19], *Borassus flabellifer* [20], *Solanum lycocarpum* [21], *Sinocrassula indica* [22], *Shorea roxburghii* [23], *Cistanche tubulosa* [24, 25], and *Helichrysum arenarium* [26], which could suppress elevated blood glucose levels in sugar-loaded rats and/or mice models. Our search for antidiabetic principles from plants of the genus *Salacia* began following the discovery of the suppressive effects of a methanol extract prepared from the roots and stems of *S. reticulata* (collected in Sri Lanka) on elevated blood glucose levels in maltose- and sucrose-loaded rats at a dose of 50 mg/kg (*p.o.*); the extract did not affect glucose-loaded rats up to 200 mg/kg (*p.o.*) [2, 28]. In addition, the extract did not exhibit hypoglycemic activity in alloxan-induced insulin-dependent diabetic mice following a single administration of 3000 mg/kg (*p.o.*) [2, 28]. To characterize the mechanism underlying the suppression of postprandial glucose activity, the inhibitory effects on small intestinal *a*-glucosidases, such as maltase and sucrase, were evaluated using rat small intestinal brush border membrane vesicles as an enzymatic mixture. Consequently, the extract inhibited the enzymatic activity of both maltase (IC_50_ = 42 μg/mL) and sucrase (IC_50_ = 32 μg/mL). Thus, the *S. reticulata* extract was characterized as having *α*-glucosidase inhibitory activity, which inhibited the hydrolysis of oligosaccharides, such as maltose and sucrose, to glucose [2, 27, 28].

### Unique thiosugar sulfonium sulfates, salacinol (1) and kotalanol (3), were isolated as the active principles by bioassay-guided separation using the maltase and sucrase inhibitory activities

**Fig. 3:**
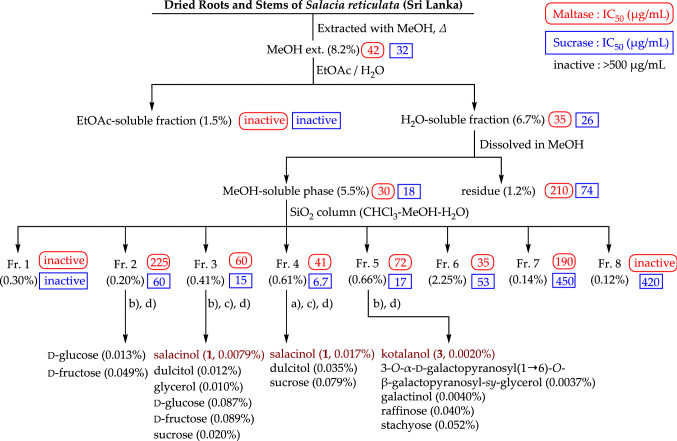
Bioassay-guided separation from *S. reticulata* using the maltase and sucrase inhibitory activities. *conditions:* **a** ODS column (MeOH–H_2_O); **b** NH column (CH_3_CN–H_2_O); **c** HPLC [detection: refractive index, column: Shodex SUGAR SC1011 (Ca^2+^) and SUGAR SP0810 (Pb^2+^) for ligand-exchange chromatography, mobile phase: H_2_O, column temperature: 80 °C]; **d** HPLC [detection: refractive index, column: YMC-Pack Polyamine II, mobile phase: CH_3_CN–H_2_O solvent system]. Reproduced in part with permission from *Bioorg. Med. Chem.*, **10**, 1547–1554. Copyright [2002] Elsevier

Our first experiments evaluated the fractionation and isolation of the antidiabetic principles from extracts, including solvent distribution and filtration, column chromatography, and preparative HPLC; these procedures are summarized in Fig. 3. Thus, the active MeOH-soluble fraction (IC_50_ = 30 μg/mL for maltase and 18 μg/mL for sucrase) was subjected to normal-phase silica gel column chromatography to obtain eight fractions. Among these, fractions 3–6 presented maltase inhibitory activity (IC_50_ = 35–72 μg/mL), while fractions 2–5 presented sucrase inhibitory activity (IC_50_ = 6.7–60 μg/mL). Further separation and purification procedures using ODS and NH column chromatography, and finally preparative HPLC, isolated the active constituents salacinol (**1**) [27, 28] and kotalanol (**3**) [29] (Fig. 2), along with several sugars and sugar alcohols, including d-glucose, d-fructose, dulcitol, glycerol, sucrose, 3-*O*-*α*-d-galactopyranosyl(1 → 6)-*O*-*β*-galactopyranosyl-*sy*-glycerol, galactinol, and stachyose. Because the oligosaccharides as substrate for the enzymatic activities and d-glucose were obtained from the active fractions such as fractions 2–6, the condensation of the maltase and sucrase inhibitory activities of those fractions were not observed as much as the condensation of the active isolates (**1** and **3**). The structure of salacinol (**1**) was elucidated based on physicochemical evidence, including the NMR assignments, using several spectroscopy measurements and application of the deuterium shift rule to facilitate the locations of free hydroxy groups. Alkaline treatment of salacinol (**1**) with sodium methoxide gave 1-deoxy-4-thio-d-arabinofuranose (**1a**), which was identical to the synthesis from d-xylose. Finally, the absolute stereostructure was elucidated by X-ray crystallographic analysis, which showed that the unique spiro-like configuration of the inner salt was comprised of 1-deoxy-4-thio-d-arabinofuranosyl sulfonium cation and 1′-deoxy-d-erythrosyl-3′-sulfate anion [27, 28]. The stereostructure of kotalanol (**3**) was also characterized [29, 30]. To our knowledge, 5-thio-d-mannose was hitherto isolated from a marine sponge as the only naturally occurring thiosugar [31], and these compounds (**1** and **3**) are the first examples of sulfonium-type thiosugars in nature.

### A series of other thiosugar sulfonium constituents, neosalacinol (2), neokotalanol (4), and related isolates (5–8) from plans of the genus *Salacia*

**Fig. 4:**
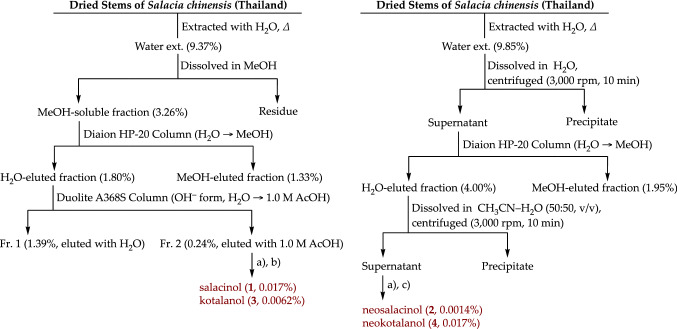
Practical isolation protocols of the principal thiosugar sulfoniums (**1**–**4**) from the stems of *S. chinensis*. *conditions:* **a** NH column (CH_3_CN–H_2_O), **b** HPLC [detection: refractive index, column: Cosmosil Sugar-D, mobile phase: CH_3_CN–H_2_O solvent system], and **c** HPLC [detection: refractive index, column: Daisopak-SP-120-5-ODS-BP, mobile phase: H_2_O and/or 0.1% (v/v) aqueous AcOH]. Reproduced in part with permission from *J. Pharm. Biomed. Anal.*, **52**, 770–773. Copyright [2010] Elsevier and *J. Nat. Med.*, **65**, 142–148. Copyright [2011] Springer Nature

As described above, a unique thiosugar sulfonium sulfate salacinol (**1**), which had a sulfated C4 polyol side chain connected at the sulfonium moiety, was first isolated from the methanol extract of *S. reticulata* and subject to structure determination, in 1997 [27, 28]. The related analog of **1** elongated the polyol side chain to C7, kotanlanol (**3**), and was isolated from the same plant resource in 1998 [29, 30]. Subsequently, **1** and **3** were isolated from the 80% aqueous methanol extract of *S. oblonga* in 1999 [32] and from the methanol extract of *S. chinensis* (*syn. S. pronoides*) in 2008 [33]. From *S. chinensis*, other related thiosugar sulfonium sulfates, ponkoranol (**5**) and salaprinol (**7**), were obtained, which have a sulfated C6, and C3 polyol side chains connected at the sulfonium moiety, respectively [33]. In addition, the desulfonated analogs of these sulfate ester constituents (**1**, **3**, **5**, and **7**), named neosalacinol (**2**) [35], neokotalanol (**4**) [35], neoponkoranol (**6**) [36, 37], and neosalaprinol (**8**) [36, 37], which presented higher polarity than each corresponding 3′-*O*-sulfate ester, were also obtained from the hot water extracts of genus *Salacia* plants. Thus, we optimized the practical isolation protocol by using the stems of *S. chinensis* originating in Thailand, and performing hot water extraction. The results demonstrated that we have established practical isolation procedures for **1**–**4**, as shown in Fig. 4 [34].

### Thiosugar sulfoniums (1–6) as a novel class of *α*-glucosidase inhibitors

As shown in Table 1, salacinol (**1**) and kotalanol (**3**) were found to inhibit maltase, sucrase, and isomaltase inhibitory activities against rat small intestinal *α*-glucosidase (IC_50_ = 6.0, 1.3, and 1.3 μM for **1**; 2.0, 0.43, and 1.8 μM for **3**, respectively) [37]. The maltase inhibitory activities of **1** were weaker than those of acarbose and voglibose (1.7 and 1.3 μM, respectively) and equivalent to that of miglitol (8.2 μM). Regarding sucrase inhibitory activity, **1** (1.3 μM) demonstrated equivalence to acarbose (1.5 μM), whereas the isomaltase inhibitory activity was more potent than those of acarbose, voglibose, and miglitol (1.5, 0.22, and 0.43 μM, respectively). However, the common thiosugar moiety 1-deoxy-4-thio-d-arabinofuranose (**1a**) did not present this activity (each IC_50_ value > 400 μg/mL for maltase, sucrase, and isomaltase). These data indicated that the sugar alcohol side chain connecting the sulfonium parts was essential for the activity. To examine how **1** and **3** inhibited maltase, sucrase, and isomaltase, small intestinal brush border membrane vesicles were incubated with increasing concentration of maltose (3.1–37 mM, *K*_m_ = 2.7 mM), sucrose (4.6–37 mM, *K*_m_ = 20 mM), and isomaltose (0.46–3.7 mM, *K*_m_ = 4.5 mM). The results plotted according to the Lineweaver–Burk revealed fully competitive inhibition on each *α*-glucosidase, and the *K*_i_ values were 0.31, 0.32, and 0.47 μg/mL for **1**; and 0.23, 0.18, and 1.8 μg/mL for **3**, respectively [28, 29].

**Table 1:** IC_50_ values of thiosugar sulfoniums (**1**–**8** and **1a**), acarbose, voglibose, miglitol, and 1-deoxynojirimycin against α-glucosideses from rat small intestine, *Saccharomyces cerevisiae*, and *Bacillus stearothermophilus*

	IC_50_ (*μ*M) [(*μ*g/mL)]						
	Rat^*a*^			*Saccharomyces cerevisiae*^*b*^		*Bacillus stearothermophilus*^*c*)^	
	Maltase	Sucrase	Isomaltase	Maltase	Sucrase	Maltase	Sucrase
Salacinol (**1**)	6.0 [2.0]	1.3 [0.42]	1.3 [0.44]	> 100	> 100	> 100	> 100
Neosalacinol (**2**)	22.2 [5.7]	2.5 [0.65]	0.68 [0.17]	> 100		> 100	
Kotalanol (**3**)	2.0 [0.86]	0.43 [0.18]	1.8 [0.78]	> 100		> 100	
Neokotalanol (**4**)	1.6 [0.54]	1.5 [0.53]	0.46 [0.16]	> 100	> 100	> 100	> 100
Ponkoranol (**5**)	5.6 [2.2]	0.41 [0.16]	4.6 [1.8]	> 100		> 100	
Neoponkoranol (**6**)	5.1 [1.6]	1.0 [0.32]	1.4 [0.43]	> 100		> 100	
Salaprinol (**7**)	> 329 [> 100]	> 329 [> 100]	14 [4.4]				
Neosalaprinol (**8**)	> 444 [> 100]	90 [20]	6.5 [1.5]				
**1a**	[> 400]	[> 400]					
Acarbose	1.7 [1.1]	1.5 [1.0]	645 [417]	> 100	> 100	0.20 [0.13]	0.021 [0.014]
Voglibose	1.3 [0.34]	0.22 [0.060]	2.2 [0.58]	> 100	> 100	> 100	> 100
Miglitol	8.2 [1.7]	0.43 [0.090]	4.6 [0.96]	> 100	> 100	> 100	> 100
1-Deoxynojirimycin	0.67 [0.11]	0.12 [0.020]	0.26 [0.042]	> 100	> 100	84.3 [13.8]	2.4 [0.39]

*α*-*Glucosidase inhibitory activity: *^a^Rat small intestinal brush border membrane vesicles, ^b^*Saccharomyces cerevisiae* (purchased from Sigma-Aldrich Co., LLC, St. Louis, USA), or ^c^*Bacillus stearothermophilus* (purchased from Sigma-Aldrich) in 0.1 M maleate buffer (pH 6.0) was prepared as an enzyme solution, respectively. A substrate solution in the maleate buffer (maltose or sucrose: 74 mM; isomaltose: 7.4 mM, 50 μL), the test sample solution (25 μL), and the enzyme solution (25 μL) were mixed at 37 °C for 30 min and then immediately heated in boiling water for 2 min to stop the reaction. The glucose concentrations were determined using the glucose-oxidase method. The IC_50_ value was determined graphically by plotting the percent inhibition *vs*. log of the test compound. Each value represents the mean of two–four experiments. Commercial acarbose, voglibose, miglitol, and 1-deoxynojirimycin were purchased from FUJIFILM Wako Pure Chemicals Co. (Osaka, Japan)

Reproduced in part with permission from *Phytochem. Anal.*, **25**, 544–550. Copyright [2014] Jhon Wiley & Sons, Ltd

Furthermore, we have also evaluated the inhibitory activities of the active sulfoniums (**1**–**6**) against human intestinal maltase [38]. As shown in Table 2, 1 (IC_50_ = 4.9 μM), **2** (9.0 μM), **3** (3.9 μM), **4** (3.9 μM), **5** (5.0 μM), and **6** (4.0 μM) inhibited the enzymatic activities of maltase, with almost equivalent activity to miglitol (3.7 μM) and greater potency than acarbose (15.2 μM). According to the Lineweaver–Burk plot, inhibition was characterized as being fully competitive, and the *K*_i_ values of **1**–**6** were 0.44, 1.2, 0.32, 0.33, 0.32, and 0.70 μM, respectively.

**Table 2:** IC_50_ and *K*_i_ values of principal thiosugar sulfoniums (**1**–**6**), acarbose, voglibose, miglitol, and 1-deoxynojirimycin against human small intestinal maltase

	IC_50_ (*μ*M)	*K*_i_ (*μ*M)
Salacinol (**1**)	4.9	0.44
Neosalacinol (**2**)	9.0	1.2
Kotalanol (**3**)	3.9	0.32
Neokotalanol (**4**)	3.9	0.33
Ponkoranol (**5**)	5.0	0.32
Neoponkoranol (**6**)	4.0	0.70
Acarbose	15.2	2.6
Voglibose	1.3	0.17
Miglitol	3.7	0.57
1-Deoxynojirimycin	0.96	0.071

*Maltase inhibitory activity:* Human small intestinal microsome (batch MIC318017, purchased from BIOPREDIC International, Rennes, France) in 0.1 M maleate buffer (pH 6.0) was prepared as an enzyme solution. A substrate solution in the maleate buffer (maltose: 74 mM, 50 μL), the test sample solution (25 μL), and the enzyme solution (25 μL) were mixed at 37 °C for 30 min and then immediately heated in boiling water for 2 min to stop the reaction. The glucose concentrations were determined using the glucose-oxidase method. The IC_50_ value was determined graphically by plotting the percent inhibition *vs*. log of the test compound. Each value represents the mean of four experiments. Commercial acarbose, voglibose, miglitol, and 1-deoxynojirimycin were purchased from FUJIFILM Wako Pure Chemicals Co. (Osaka, Japan)

*Kinetic analysis:* The enzyme and test samples (1.0–4.0 μM: acarbose; **1** and **3**: 0.5–2.0 μM; **2**–**6** and miglitol: 0.25–1.0 μM; 0.10–0.40 μM: voglibose) were incubated with increasing concentrations of maltose (3.0–10.6 mM)

Reproduced in part with permission from *Nutrients*, **7**, 1480–1493. Copyright [2015] MDPI

To date, several research groups have performed synthetic and structure–activity relationship (SAR) studies of salacinol (**1**) and related analogues regarding *α*-glucosidase inhibitory activity [39–55]. We are also performing subsequent studies on the total syntheses of these sulfonium constituents (**1–8**) and their highly active analogues, as well as more detailed SAR studies [38, 56–62]; those data will be summarized separately.

### Terpenoid and polyphenol constituents with aldose reductase inhibitory activity

**Fig. 5:**
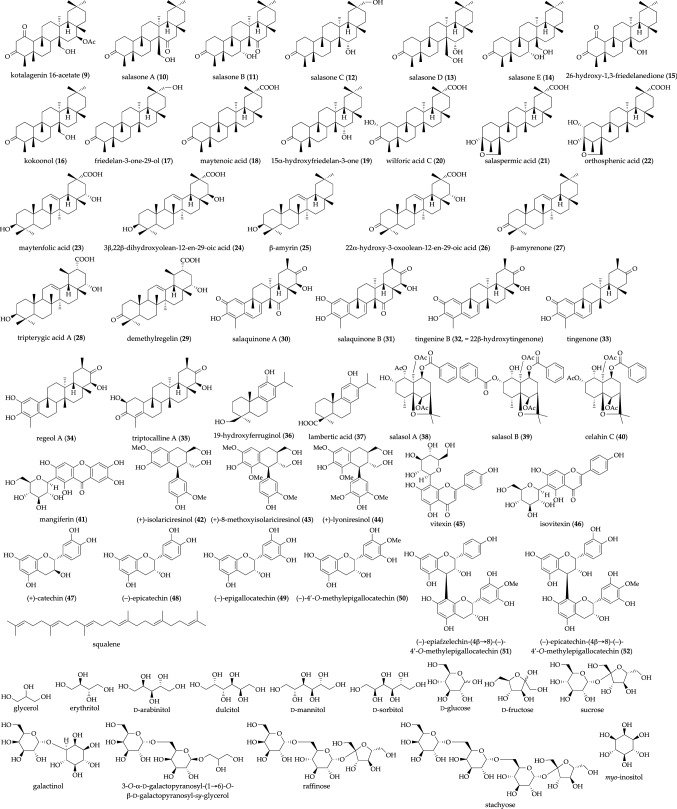
Terpenoid, polyphenol, and polyol constituents isolated from plants of the genus *Salacia*

Aldose reductase is a key enzyme that catalyzes the reduction of glucose to sorbitol in the polyol processing pathway. In normal tissue, aldose reductase has low substrate affinity to glucose, such that the conversion of glucose to sorbitol is little catalyzed. However, in diabetes mellitus, the increased availability of glucose in insulin-insensitive tissues (*e.g.*, lens, nerve, and retina) enhances the formation of sorbitol through the polyol pathway. Sorbitol dose not readily diffuse across cell membranes and thus accumulate intracellularly. The intracellular accumulation of sorbitol has been implicated in the chronic complications of diabetes, including cataracts, neuropathy, and retinopathy. These findings suggest that aldose reductase inhibitors have the capacity to prevent and treat such diabetic complications. Previously, we reported several aldose reductase inhibitors obtained from natural resources, such as flavonoids [63–69], stilbenoids [23, 65], quinic acid derivatives [68], and phenylthanoids [24]. As a continuation of the above study, several polyphenol constituents with aldose reductase inhibitory activity from *S. reticulata* [70], *S. oblonga* [32], and *S. chinensis* [71, 72] were further explored. We also investigated the inhibitory effect of the 80% aqueous methanol extracts of *S. oblonga* and *S. chinensis* against aldose reductase (IC_50_ = 3.4 and 3.6 μg/mL, respectively) [32, 71]. As shown in Fig. 5, several terpenoids including 14 friedelane-type (**9**–**22**), five oleanane-type (**23**–**27**), two ursane-type (**28** and **29**), and six nor-friedelane-type triterpenes (**30**–**35**) and squalene, two abietane-type diterpenes (**36**, **37**), and three acylated eudesmane-type sesquiterpenes (**38**–**40**), polyphenols including an xanthone, mangiferin (**41**), three lignans (**42**–**44**), two flavones (**45** and **46**), and six flavan-3-ols (**47**–**52**), 13 sugar derivatives, and a cyclitol, *myo*-inositol, were isolated from genus *Salacia* plants [28, 32, 33, 70–73]. Oleanane-type triterpenes, feidelane-3-one-29-ol (**17**, IC_50_ = 98 μM), maytenfolic acid (**23**, 72 μM), and 3*β*,22*β*-dihydroxyolean-12-en-29-oic acid (**24**, 26 μM), norfriedelane-type tritepenes, tingrnine B (**32**, 7.0 μM), tingenone (**33**, 13 μM), regeol A (**34**, 30 μM), and triptocalline A (**35**, 14 μM), an acylated eudesmane sesquiterpene celahin C (**38**, 95 μM), and a xanthone mangiferin (**41**, 3.2 μM), were found to exhibit the inhibitory effects of the constituents on aldose reductase (Table 3) [72]. Among those, mangiferin (**41**) was suggested to be the most active constituent in the extract of plants from the genus *Salacia* against aldose reductase [70]. However, the inhibitory activity was moderate compared with that of a clinically used aldose reductase inhibitor epalrestat (0.0072 μM); therefore, the contribution of aldose reductase inhibitory activity on the antidiabetic effect of plants from the genus *Salacia* is limited.

**Table 3:** Inhibitory effects of constituents from plants of the genus *Salacia* on rat lens aldose reductase

	IC_50_ (*μ*M)
Friedelan-3-one-29-ol (**17**)	98
Maytenfolic acid (**23**)	72
3*β*,22* β*-Dihydroxyolean-12-en-29-oic acid (**24**)	26
Tingenine B (**32**)	7.0
Tingenone (**33**)	13
Regeol A (**34**)	30
Triptocalline A (**35**)	14
Celahin C (**38**)	95
Mangiferin (**41**)	3.2
Epalrestat	0.0072

*Aldose reductase inhibitory activity:* The supernatant fluid of a rat lens homogenate was used as a crude enzyme. The incubation mixture contained 180 mM Na, K-phosphate buffer (pH 7.0), 100 mM Li_2_SO_4_, 0.03 mM NADPH, 1 mM dL-glyceraldehyde as a substrate, and 100 μL of enzyme fraction, with or without 25 μL of sample solution, in a total volume of 0.5 mL. The reaction was initiated by the addition of NADPH at 30 °C. After 30 min, the reaction was stopped by the addition of 150 μL of 0.5 M HCl. Then, 0.5 mL of 6 M NaOH containing 10 mM imidazole was added, and the solution was heated at 60 °C for 20 min to convert NADP to a fluorescent product. Fluorescence was measured using a fluorophotometer (luminescence spectrometer LS50B, Perkin-Elmer, UK) at an excitation wavelength of 360 nm and an emission wavelength of 460 nm. Each test sample was dissolved in DMSO. Measurements were made in duplicate, and the IC_50_ value was determined graphically by plotting the percent inhibition *versus* log of the test compound. An aldose reductase inhibitor epalrestat was used as a reference compound

Reproduced with permission from *J. Nat. Prod.*, **66**, 1191–1196. Copyright [2003] ACS

## Quantitative evaluation of principal sulfonium constituents as characteristic marker molecules

**Fig. 6:**
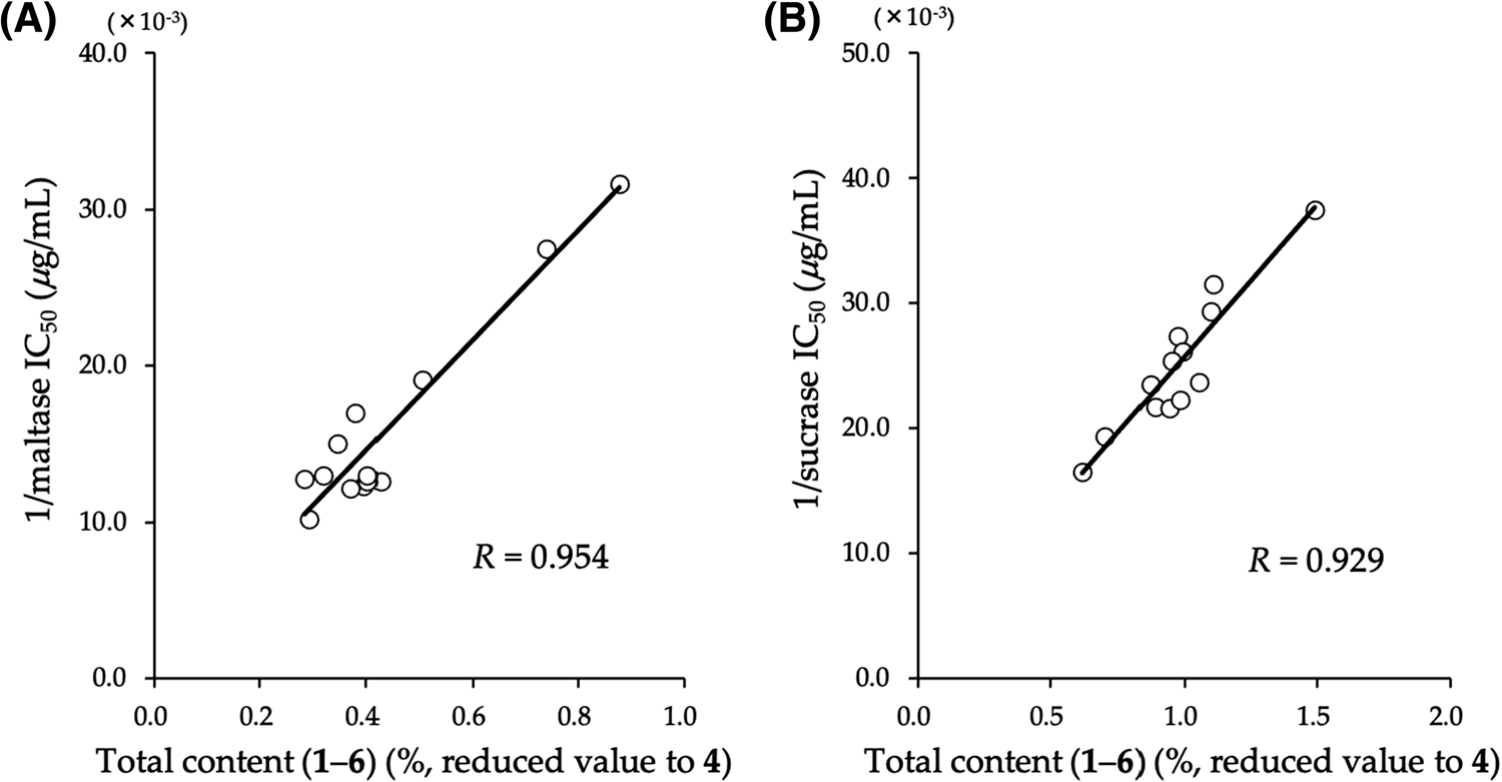
Correlations between maltase and sucrase inhibitory activities and total content of six thiosugar sulfoniums (**1**–**6**). Total contents (%) of the six thiosugar sulfoniums (**1**–**6**) are presented in values reduced to the content of neokotalanol **(4**), calculated based on the ratio of IC_50_ values (*μ*g/mL) of **1**–**6** against those of (**a**) maltase or (**b**) sucrase

As discussed, we previously investigated the suppressive effects of the methanol extract from the roots and stems of *S. reticulata* on the elevated blood glucose levels in maltose- and sucrose-loaded rats [2, 28]. We have also demonstrated the antihyperglycemic effects of the 80% aqueous methanol extracts from *S. oblonga* and *S. chinensis,* as well as *S. reticulata* using the same sugar-loaded animal models [2, 71]. Based on these findings, interest in *Salacia* as a possible nutraceutical product for patients with diabetes and/or prediabetes is increasing. Thus, there has been a high demand for efficient quality control measures to ensure the authenticity and active contents of these products, and to verify the claims on product labels. Therefore, to evaluate the quality of *Salacia* extracts for antidiabetic effects, quantitative analyses of sulfonium constituents (**1**–**8**) have been developed as two separate protocols using LC–MS. The sulfonated derivatives (**1**, **3**, **5**, and **7**) were obtained using an Asahipak NH2P-50 column (Showa Denko K.K., Tokyo, Japan) with an acetonitrile–water solvent system (78:22, v/v) as a mobile phase, associated with negative-ion electrospray ionization mass (ESI–MS) sources (*m/z* 333, 423, 393, and 303 [M – H]^–^, respectively) [34, 37]. The de-*O*-sulfonate derivatives (**2**, **4**, **6**, and **8**) were established by ion pair chromatography using an ODS column with 5 mM aqueous undecafluorohexanoic acid–MeOH (99:1, v/v) as the mobile phase and positive-ion ESI–MS measurement (*m/z* 255, 345, 319, and 225 [M]^+^, respectively) [35, 37]. Using the established protocols, a variety of *Salacia* samples collected in different geographical regions (*e.g.,* Sri Lanka, India, and Thailand), as well as their distribution in each part of the plant, including the stems, roots, leaves, and fruit, were evaluated. The distribution of sulfoniums (**1**–**8**) in the stems and roots of these plants differed between the collection areas. Among these, neokotalanol (**4**) was the major compound in samples from Thailand, whereas salacinol (**1**) was the major compound in samples from Sri Lanka and India. Regarding differences in the characteristic distributions between plant parts, the sulfoniums were only present in trace amounts in the leaf and fruit parts. [34, 35, 37]. An effort was made to discriminate the *Salacia* plant species based on the DNA sequence of the internal transcribed spacer (ITS) region in the nuclear ribosomal RNA gene in an authentic specimen, and a genotype characteristic of *S. chinensis*, which is distinguishable from those of *S. reticulata* and *S. oblonga* was identified [74]. Correlations between the total content of four principal sulfoniums (**1**–**4**) and the maltase and sucrase inhibitory activities (1/IC_50_) of the corresponding extracts from the stems of *S. chinensis* were plotted. Strong correlations were observed between the total content (%, reduced value to **4**) and inhibitory activity (*R* = 0.959 for maltase and 0.795 for sucrase) [35]. Furthermore, when ponkoranol (**5**) and neoponkoranol (**6**) were plotted in addition to total sulfonium (**1**–**6**), these correlations were found to be stronger and almost fully explained both the maltase (*R* = 0.954) and the sucrase (*R* = 0.929) inhibitory activities of the extract (Fig. 6). Thus, these practical LC–MS methods for the quantitative determination of sulfoniums with potent α-glucosidase inhibitory activity could be readily utilized for the evaluation of genus *Salacia* plants.

## Evaluation of hot water extract from the stems of *S. chinensis* (SCE) as a functional food material for improving the effects on blood glucose and HbA1c levels in animal models

**Fig. 7:**
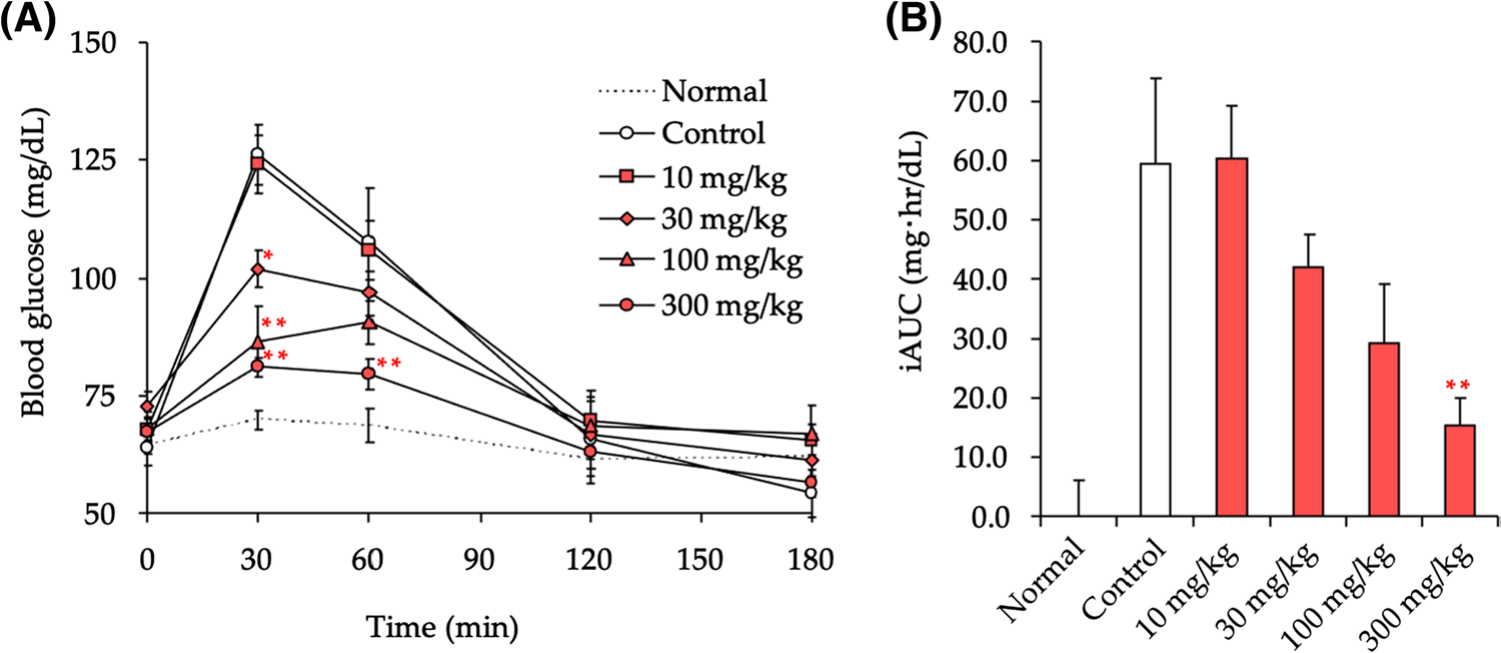
Effect of SCE on blood glucose levels in starch-loaded rats. Male SD rats (5-week-old, Kiwa Laboratory Animals, Ltd., Wakayama, Japan) were housed for 1 week in meal cages. Animals were fasted overnight for 20 h, but allowed water ad libitum, and the rats were then administered a 5% (w/v) α-starch solution (1 g/kg) orally with or without a sample (SCE, 10–300 mg/kg) using a stomach tube. At 0, 30, 60, 120, and 180 min after the administration of α-starch, blood samples were taken from the tail vein and immediately used to measure blood glucose via the glucose-oxidase method. As a baseline, distilled water was administrated to rats in the Normal group. Median effective dose (ED_50_) was determined by plotting the inhibition rate of incremental AUC_0-120 min_ (i AUC_0-120 min_; the AUC above baseline) *versus* corresponding inhibitor dosage. Each value represents the mean ± S.E.M. (*n* = 8). Significantly different from the control: ^*^p < 0.05, ^**^p < 0.01. Reproduced with permission from *Nutrients*, **7**, 1480–1493. Copyright [2015] MDPI

In Japan, the government can label two types of food products with certain health claims: Foods for Specified Health Uses (FOSHU) and Foods with Function Claims (FFC) [75–81]. Due to the increasing interest in plants of the genus *Salacia* as a possible food product with health claim for individuals with prediabetes and/or those with high blood glucose levels, we examined the suppressive effects of the hot water extract from the stems of *S. chinensis* (SCE). Strong correlations have been observed between the total content of four principal thiosugar sulfoniums (**1**–**4**) and the *a*-glucosidase inhibitory activity (*vide supra*), on postprandial blood glucose levels in starch-loaded rats. As shown in Fig. 7, SCE significantly suppressed the increase in blood glucose levels in a dose-dependent manner (30–300 mg/kg, *p.o.*), with an ED_50_ value of 94.0 mg/kg. Among the sulfonium constituents, salacinol (**1**), kotalanol (**3**), and neokotalanol (**4**) were also evaluated using the in vivo assay, with ED_50_ values of > 2.06, 0.62, and 0.54 mg/kg, respectively [38].

**Fig. 8:**
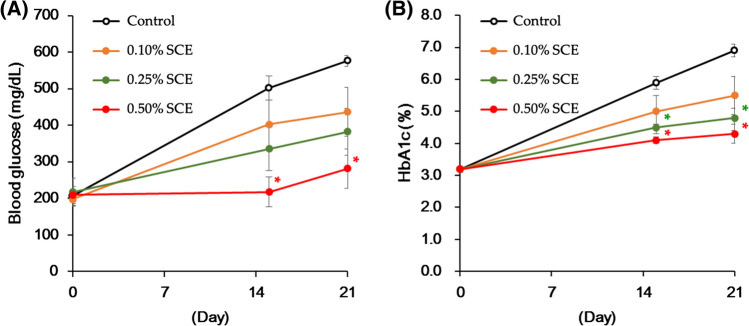
Effect of chronic administration of SCE on blood glucose and HbA1c levels in CE-2 diet-fed KK-A^y^ mice. Male KK-A^y^ mice (5-week-old, CLEA Japan, Inc., Tokyo, Japan) were housed for 1 week in individual meal cages. These mice were divided into four groups based on body weight, blood glucose, and HbA1c levels. Mice in the control group were fed a standard diet (CE-2, CLEA Japan, Inc.) and those in the SCE-treated groups were fed diets supplemented with 0.10, 0.25, and 0.50% (w/w) SCE, respectively. On day 15 and at the end of the treatment period, blood samples were taken from the tail vein under non-fasting conditions. Blood glucose and HbA1c levels were measured using the glucose-oxidase method and a DCA Vantage Analyzer™ (Siemens, New York, USA), respectively. Each value represents the mean ± S.E.M. (*n* = 6). Significantly different from the control: ^*^p < 0.05, ^**^p < 0.01. Reproduced with permission from *Nutrients*, **7**, 1480–1493. Copyright [2015] MDPI

Next, the effects of 3-weeks’ administration of SCE on postprandial blood glucose and HbA1c levels were evaluated in a typical model of type 2 diabetes mellitus (KK-A^y^ mice). As shown in Fig. 8, feeding animals a CE-2 diet containing 0.25 and/or 0.50% (w/w) SCE significantly suppressed the increase in both blood glucose and HbA1c levels without significant changes in body weight and food intake. Furthermore, a glucose tolerance test (2 g/kg) was performed following continuous administration of an AIN93M purified diet containing 0.12% (w/w) SCE to glucose-loaded KK-A^y^ mice for 27 days. The results showed that SCE significantly suppresses the elevation in blood glucose. Thus, SCE exerted antidiabetic effects by both inhibiting the increase in postprandial blood glucose levels and improving glucose tolerance [38].

To verify whether the suppressive effects of SCE on HbA1c levels were due to the presence of *α*-glucosidase inhibitors, we performed similar chronic experiments using a customized diet, in which all the digestible glucides in AIN93M (AIN93M/Glc) were substituted by d-glucose. There were no significant differences in HbA1c levels in KK-A^y^ mice fed a customized (AIN93M/Glc) or standard (AIN93M purified) diet supplemented with 0.30% SCE for 14 days compared with the corresponding control group. These results indicate that the antidiabetic effect of SCE is due to the potent *α*-glucosidase inhibitory activity of its active constituents, which are characteristic sulfoniums, including salacinol (**1**), neokotalanol (**4**), and their related analogues isolated from genus *Salacia* plants [38].

**Fig. 9:**
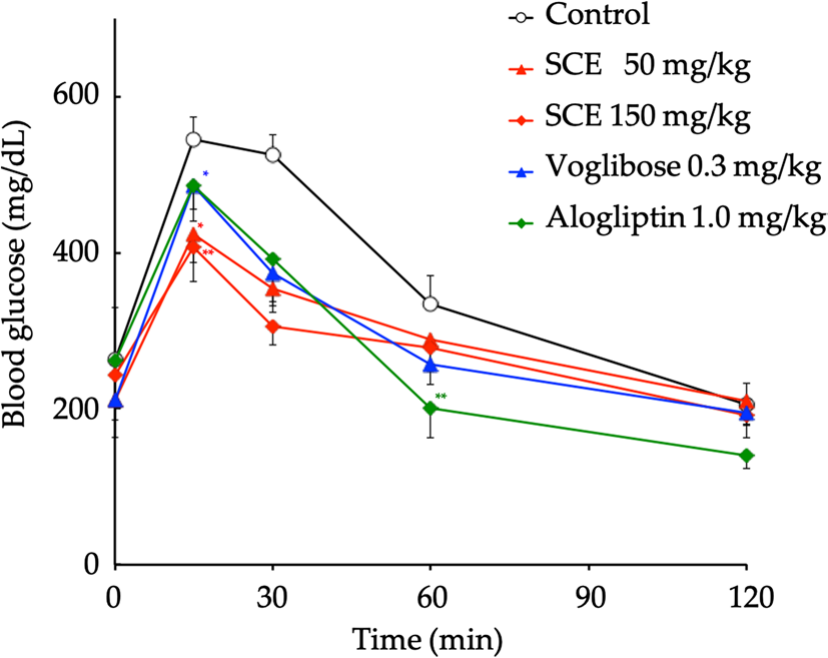
Effects of SCE, voglibose, and alogliptin on blood glucose levels in Ensure H^®^-loaded *ob/ob* mice. Male B6.Cg-Lep^ob^/J (*ob/ob*) mice (6-week-old, Charles River Laboratories Japan, Inc., Yokohama, Japan) were housed for 1 week in individual meal cages. Mice were fasted overnight for 20 h, but allowed water ad libitum. Then, the mice were orally administered an enteral nutrient Ensure H^®^ [10 mL/kg (per 10 mL energy: 15 kcal; dextrin: 1668 mg; sucrose: 392 mg)] with or without a sample (SCE: 50 or 150 mg/kg) using a stomach tube. At 0, 15, 30, 60, and 120 min after administration, blood samples were taken from the tail vein and immediately used to measure blood glucose via the glucose-oxidase method. Each value represents the mean ± S.E.M. (*n* = 6). Significantly different from the control: ^*^p < 0.05, ^**^p < 0.01. Reproduced with permission from *J. Nat. Med.*, **73**, 584–588. Copyright [2019] Springer Nature

**Fig. 10:**
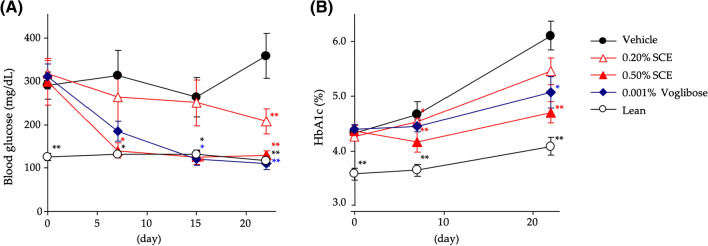
Effect of SCE and voglibose on blood glucose and HbA1c levels after 23 days administration in CE-2 diet-fed *ob/ob* mice. Male B6.Cg-Lep^ob^/J (*ob/ob*) mice (6-week-old, Charles River Laboratories Japan, Inc., Yokohama, Japan) were housed for 1 week in individual meal cages. These mice were divided into five groups based on body weight, blood glucose, and HbA1c levels. Mice in the control group were fed a standard diet of CE-2. Mice in the SCE-treated and positive control groups were fed the same diet supplemented with 0.20 and 0.50% (w/w) of SCE, respectively. Mice in the positive control group were fed the same diet supplemented with 0.001% of voglibose. On days 7, 15, and 22, blood glucose and HbA1c levels were measured using the glucose-oxidase method and a DCA Vantage Analyzer™ (Siemens, New York, USA), respectively. Each value represents the mean ± S.E.M. (*n* = 6). Significantly different from the control: ^*^p < 0.05, ^**^p < 0.01. Reproduced with permission from *J. Nat. Med.*, **73**, 584–588. Copyright [2019] Springer Nature

In addition, we examined the antidiabetic effects of SCE and its principal thiosugar sulfonium, neokotalanol (**4**), using genetically hyperglycemic model *ob/ob* mice, which are grossly overweight, hyperphagic, obese, hyperinsulinemic, and hyperglycemic, and used as models of diabetes with obesity [82]. Thus, administration of a single-dose of SCE significantly suppressed the elevated blood glucose in enteral nutrient Ensure H^®^ (10 mL/kg, Abbott Japan Co., Ltd., Tokyo, Japan)-loaded *ob*/*ob* mice in a dose-dependent manner (50–150 mg/kg *p.o.*) (Fig. 9). Thus, the suppressive curve of the blood glucose elevation of SCE was similar to that of a clinical *α*-glucosidase inhibitor voglibose, but dissimilar to that of a clinical dipeptidyl peptidase-4 (DPP-4) inhibitor, alogliptin. Furthermore, continuous administration of 0.20 and 0.50% (w/w) SCE in CE-2 diet-fed *ob*/*ob* mice for 23 days significantly suppressed the increase in both blood glucose and HbA1c levels in a dose-dependent manner (Fig. 10). Notably, the water intake of mice in the SCE-treated groups was lower than that of mice in the control group during the administration period [average intake per day: 0.20% SCE group (7.7 ± 1.2 g), 0.50% SCE group (6.2 ± 0.6 g), and Control group (11.5 ± 2.0 g)], which was similar to that of mice treated with 0.001% (w/w) voglibose (5.6 ± 0.5 g). These results suggest that SCE has a beneficial effect on polydipsia with diabetes mellitus.

Similarly, the antidiabetic effects of neokotalanol (**4**), one of the highest contributing principles based on its potent *α*-glucosidase inhibitory activity and high content in SCE, were evaluated by evaluating the blood glucose and HbA1c levels of *ob/ob* mice following 20-day continuous administration. As show in Table 4, administration of the diet containing 0.0003% of neokotalanol (**4**) was found to significantly suppress the increase in HbA1c levels without causing changes in body weight. Consequently, the potent *α*-glucosidase inhibitor neokotalanol (**4**) was identified as one of the active constituents hampering the progress of diabetes in obese-hyperglycemic *ob/ob* mice.

**Table 4:** Effects of SCE, neosalacinol (**4**), and voglibose on food and water intakes, body weight, and HbA1c levels after 20 days of administration in AIN-93 M purified diet-fed *ob*/*ob* mice

	Dose	Food intake	Water intake	Body weight (g)							
	(%)	(g/day, average)		Day 0	Day 3	Day 6	Day 9	Day 12	Day 15	Day 18	Day 20
Control	–	3.5 ± 0.1	4.0 ± 0.7	32.9 ± 0.6	35.6 ± 0.7	36.4 ± 0.4	36.9 ± 0.5	38.1 ± 0.4	39.2 ± 0.5	40.3 ± 0.4	41.0 ± 0.4
SCE	0.05	3.6 ± 0.1	2.5 ± 0.1^**^	33.4 ± 0.5	36.1 ± 0.3	36.8 ± 0.2	37.4 ± 0.2	38.6 ± 0.3	39.3 ± 0.2	40.8 ± 0.3	41.3 ± 0.3
Neokotalanol (4)	0.0003	3.5 ± 0.2	2.5 ± 0.2^**^	32.2 ± 0.5	35.1 ± 0.4	35.8 ± 0.4	36.0 ± 0.4	37.2 ± 0.4	37.8 ± 0.4	39.4 ± 0.4	40.1 ± 0.4
Voglibose	0.0001	4.5 ± 0.2	3.3 ± 0.2	33.0 ± 0.5	36.2 ± 0.4	36.3 ± 0.5	37.1 ± 0.3	38.0 ± 0.4	39.0 ± 0.2	40.2 ± 0.3	40.8 ± 0.4

	Dose	HbA1c (%)	
	(%)	Day 0	Day 20
Control	–	6.1 ± 0.0	6.5 ± 0.2
SCE	0.05	6.1 ± 0.1	6.0 ± 0.1*
Neokotalanol (**4**)	0.0003	5.9 ± 0.1	5.9 ± 0.1**
Voglibose	0.0001	5.9 ± 0.1	6.0 ± 0.1*

*Effects on HbA1c levels of AIN-93 M purified diet-fed ob/ob mice following 20 days of administration:* Male B6.Cg-Lep^ob^/J (*ob/ob*) mice (6-week-old, Charles River Laboratories Japan, Inc., Yokohama, Japan) were housed for 1 week in individual meal cages. These mice were divided into four groups based on body weight, blood glucose, and HbA1c levels. Mice in the control group was fed a standard AIN-93 M purified diet. Mice in the SCE-, neokotalanol (**4**)-, and voglibose-treated groups were fed the same diet supplemented with 0.05, 0.0003, and 0.0001% (w/w) of the respective treatments. On days 0, 3, 6, 9, 12, 15, 18, and 20 (end of the treatment period), their body weights were measured. On days 0 and 20, the HbA1c levels were measured using Quo-Lab (Nipro, Osaka, Japan)

Each value represents the mean ± S.E.M. (*n* = 6)

Significantly different from the control: *p < 0.05, **p < 0.01

Reproduced with permission from *J. Nat. Med.*, **73**, 584–588. Copyright [2019] Springer Nature

## Mangiferin (41) is a promising marker molecule for the antidiabetic effect of plants in the genus *Salacia*

A xanthone *C*-glycoside mangiferin (**41**), originally obtained from the stem bark of mango tree (*Mangifera indica* L.) [83–85], was isolated from plants of the genus *Salacia* as a moderate aldose reductase inhibitor (*vide supra*) and reported to exert a hypoglycemic effect in KK-A^y^ mice [2, 86]. Thereafter, mangiferin (**41**) has attracted attention as a bio-functional molecule for its antidiabetic [83, 84, 87–89], antioxidant [83, 84, 90–92], antibacterial, antiviral [84], antiparasitic [84], antiinflammatory [83, 84, 90–94], and anticancer [83, 84, 95, 96] activities. These findings indicate that mangiferin (**41**) may be a possible marker molecule for the antidiabetic activity of plants from the genus *Salacia*. Therefore, simultaneous quantitative determination of polyphenol constituents, including mangiferin (**41**), by LC–MS was performed to further evaluated plants from the genus *Salacia* [97]. The results showed that the mangiferin (**41**) content in plants of the genus *Salacia,* such as *S. reticulata*, *S. oblonga*, and *S. chinensis,* from different regions were higher in the root part than in the corresponding stem part. Among the root part, the inner root bark was found to possess the richest content of mangiferin (**41**).

## Safety profiles

Extracts from plants of the genus *Salacia* have been found to have good safety profiles in animal models, such as rats, mice, guinea pigs, and horses, and also in healthy adults, and in patients with borderline diabetes and type 2 diabetes [4, 98–106]. Thus, no serious oral toxicity of *Salacia* extracts, such as aqueous extracts from *S. reticulata* and *S. oblonga,* has been observed following single-dose treatment in sub-chronic administration tests [4, 98–105]. In addition, the extract from *S. reticulata* presented no mutagenicity [98], hepatotoxicity [103], antigenicity, or phototoxicity [104]. The *S. chinensis* extract was found to exert no reproductive toxicity in SD rats, even at a high dosage level [102]. In addition, Stohs and Ray (2015) stated that no adverse effects have been reported in studies evaluating the safety of *Salacia* extracts in humans [4]. We performed two randomized double-blind placebo-controlled trials to evaluate the safety of long-term and excessive intake of the hot water extract of *S. chinensis* [106]. The subjects were healthy or had borderline diabetes with fasted blood glucose levels of 100–125 mg/dL. For the long-term intake study, 42 subjects were divided into a test group and a placebo group, and administered three tablets [containing more than 0.221 mg of neokotalanol (**4**) per tablet] per day for 12 weeks. In the excess intake study, 41 subjects were given 15 tablets per day for 4 weeks under the same conditions. No adverse effects in terms of clinical parameters were observed in either trial, confirming the safety of long-term and excessive intake of *S. chinensis* extract [106].

**Fig. 11:**
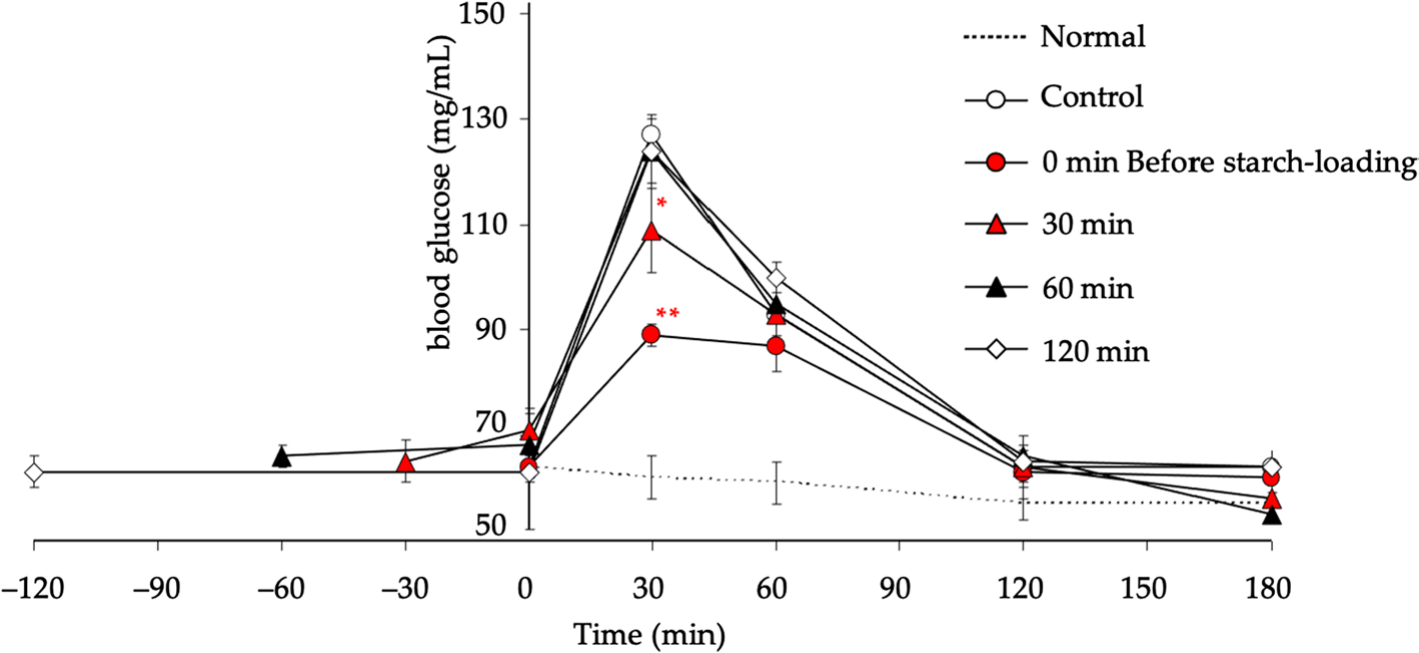
Effect of SCE on blood glucose levels in SCE-pretreated starch-loaded rats. Male SD rats (5-week-old, Kiwa Laboratory Animals, Ltd., Wakayama, Japan) were housed for 1 week in meal cages. Rats were fasted overnight for 20 h, but allowed water ad libitum. Then, the rats were orally administered SCE (75 mg/mg) using a stomach tube at 0, 30, 60, and 120 min before loading of 5% (w/v) α-starch solution (1 g/kg). At 0, 30, 60, 120, and 180 min after the administration of α-starch, blood samples were taken from the tail vein and used immediately to measure blood glucose via the glucose-oxidase method. As a baseline, distilled water was administrated to rats in the Normal group. Each value represents the mean ± S.E.M. (*n* = 8). Significantly different from the control: ^*^p < 0.05, ^**^p < 0.01. Reproduced with permission from *Nutrients*, **7**, 1480–1493. Copyright [2015] MDPI

We then evaluated the duration of the *α*-glucosidase inhibitory effect of SCE in a starch-preloaded model. Thus, starch-loaded rats for 0–120 min were administered SCE (75 mg/kg, *p.o.*) orally, and suppression of elevated blood glucose levels was subsequently observed. In the group subjected to 30 min pre-starch-loading, the increase in blood glucose level was significantly suppressed. However, no effect was observed in the group that was loaded with starch for more than 60 min before treatment, as shown in Fig. 11. Therefore, the suppressive effect of SCE against the increase in blood glucose was estimated to last for approximately 30 min after administration and then weakened over time [38].

Next, we evaluated the kinetics of the principal sulfoniums (**1**–**4**) in SCE following oral administration by examining (i) stability in an artificial gastric juice and (ii) bioavailability through the intestine using an in situ rat ligated intestinal loop model. We found that more than 96% of each sulfonium (**1**–**4**) survived following treatment at 37 °C for 1.0 h. Even after 3.0 h of treatment under the experimental conditions, more than 90% of survived, and the stability of these sulfoniums (**1**–**4**) in the artificial gastric juice was high [38]. Furthermore, these sulfoniums (**1**–**4**) were minimally absorbed in the small intestine [38]. Thus, these data indicated that the sulfoniums reached the small intestine following oral administration without being degraded by gastric juice, where they exerted inhibitory activity against *α*-glucosidase. In addition, most sulfoniums remained in the intestinal tract without being absorbed. Furthermore, SCE has no effects on reproductive outcomes in rats, even at the high dosage level of 2,000 mg/kg/day [102].

## Clinical study

Clinical trials on the aqueous extract of *S. reticulata* have demonstrated that 5 min pre-treatment with the extract (200 mg) prior to sucrose (50 g) loading suppressed postprandial blood glucose elevation in human volunteers. [107]. Additionally, an extract-containing diet (240 mg/kg/day) fed to patients with mild type 2 diabetes for 6 weeks was found to exert inhibitory effects on fasting blood glucose levels, HbA1c, and BMI in a placebo-controlled and cross-over trial [108]. The aqueous extract was also found to be an effective and safe treatment for patients with type 2 diabetes in a double-blind randomized placebo-controlled cross-over study when administered as a herbal tea containing *S. reticulata* for 3 months [109]. Finally, the extract (500 mg/day for 6 weeks) was found to improve serum lipids and glycemic control in patients with prediabetes and mild-to-moderate hyperlipidemia in a double-blind placebo-controlled, randomized trial [110]. Clinical trials have also investigated the aqueous extract of *S. oblonga*, and found it to possess suppressive effects at 500–1000 mg on postprandial plasma glucose and insulin AUC values in healthy adults [111, 112]. In addition, at 240 and 480 mg, the extract was found to possess inhibitory effects on postprandial glycemia and insulinemia in patients with type 2 diabetes after ingestion of a high-carbohydrate meal [113]. To verify the clinical effectiveness of *S. chinensis*, we evaluated the suppressive effect of SCE on postprandial hyperglycemia in human subjects. This randomized double-blind and cross-over trial was performed in 32 human volunteers with borderline diabetes and fasting blood glucose levels between 100 and 125 mg/dL. Single-dose intake of a tablet containing 100 mg of SCE with 0.221 mg neokotalanol (**4**) followed by a rice diet (200 g: containing 69.4 g of carbohydrate, 302 kcal) significantly suppressed the increase in postprandial blood glucose levels 30 min after a meal compared with the placebo. In addition, the AUC for blood glucose and serum insulin levels up to 3 h in SCE treatment group were also significantly lower than those in the placebo group [114]. Furthermore, in a placebo-controlled, randomized, double-blind cross-over trial, we recently confirmed the dose-dependent suppression of postprandial hyperglycemia, and improvement of blood glucose parameters following a single-dose of SCE (150, 300, or 600 mg). Additionally, in a placebo-controlled, randomized double-blind trial, we demonstrated that 12-week ingestion of SCE (600 mg before each of three meals daily) improved parameters related to blood glucose, such as HbA1c, glycoalbumin, and 1,5-anhydro-d-glucitol levels, and glucose tolerance after a glucose challenge [115].

## Conclusion

Since safety profiles and clinical findings associated with the antidiabetic effects of genus *Salacia* plants have been reported, several *Salacia*-containing products, which contribute to the regulation of postprandial blood glucose elevation, have been approved as FOSHU or notified as an FFC to the Consumer Affairs Agency in Japan. The evidence discussed above for the antidiabetic effects of plants from the genus *Salacia* may have contributed to the development of these functional foods. Furthermore, we hope that additional research on the genus *Salacia* as beneficial plant resources for the prevention and early treatment of diabetes, and also on their thiosugar sulfonium constituents, such as salacinol (**1**) and neokotalanol (**4**), will attract attention to these plants as promising candidates for a new class of antidiabetic agents in the future.
